# Non-invasive test using palmitate in patients with suspected fatty acid oxidation defects: disease-specific acylcarnitine patterns can help to establish the diagnosis

**DOI:** 10.1186/s13023-017-0737-7

**Published:** 2017-12-21

**Authors:** Nils Janzen, Alejandro D. Hofmann, Gunnar Schmidt, Anibh M. Das, Sabine Illsinger

**Affiliations:** 1 Screening Laboratory Hannover, Hannover, Germany; 2 Institute of Clinical Chemistry, Hannover Medical School, Hannover, Germany; 3 Center of Pediatric Surgery, Hannover Medical School and Bult Children’s Hospital, Hannover, Germany; 4 Institute of Human Genetics, Hannover Medical School, Hannover, Germany; 5 Clinic for Pediatric Kidney-, Liver- and Metabolic Diseases, Hannover Medical School, Hannover, Germany; 6 Centre for Systems Neurosciences at Veterinary School Hannover, Hannover, Germany

**Keywords:** Fatty acid oxidation, Tandem mass spectrometry, SCADD, MCADD, VLCADD, LCHADD, CTD, Palmitate, Carnitine, Whole blood sample

## Abstract

**Background:**

The aim of the present study was to establish a non-invasive, fast and robust enzymatic assay to confirm fatty acid oxidation defects (FAOD) in humans following informative newborn-screening or for selective screening of patients suspected to suffer from FAOD.

**Material/methods:**

The reliability of this method was tested in whole blood from FAOD patients with specific enzymatic defects. Whole blood samples were assayed in 30 medium chain- (MCADD, age 0 to 17 years), 6 very long chain- (VLCADD, age 0 to 4 years), 6 long chain hydroxy- (LCHAD, age 1 to 6 years), 3 short chain- (SCADD, age 10 to 13 years) acyl-CoA-dehydrogenase- and 2 primary carnitine transporter deficiencies (CTD, age 3 to 5 years). Additionally, 26 healthy children (age 0 to 17 years) served as controls. Whole blood samples were incubated with stable end-labeled palmitate; labeled acylcarnitines were analyzed by tandem mass spectrometry and compared with controls and between patient groups (Mann-Whitney Rank Sum Test). Concentrations of specific labeled acylcarnitine metabolites were compared between particular underlying MCADD- (ANOVA), VLCADD- and LCHADD- genetic variants (descriptive data analysis).

**Results:**

11 different acylcarnitines were analyzed. MCADD- (C8-, C10-carnitine, C8/C10- and C8/C4-carnitine), VLCADD- (C12-, C14:1-, C14:2-carnitine, C14:1/C12- and C14:2/C12-carnitine), LCHADD (C16-OH-carnitine) as well as CTD- deficiency (sum of all acylcarnitines) samples could be clearly identified and separated from control values as well as other FAOD, whereas the sum of all acylcarnitines was not conclusive between FAOD samples. Furthermore, C4- (SCADD), C14- (VLCADD) and C14-OH-carnitines (LCHADD) were discriminating between the FAOD groups. Metabolic parameters did not differ significantly between underlying *MCADD* variants; similar results could be observed for *VLCADD*- and *LCHADD*- variants.

**Conclusion:**

This functional method in whole blood samples is relatively simple, non-invasive and little time consuming. It allows to identify MCADD-, VLCADD-, LCHADD- and carnitine transporter deficiencies. The genetic phenotypes of one enzyme defect did not result in differing acylcarnitine patterns in MCADD, VLCADD or LCHADD in vitro.

## Background

Disorders of mitochondrial fatty acid oxidation (FAOD) are one of the most prevalent monogenic diseases with a cumulative incidence of about 1:9300 newborns [1]. Amongst this disease entity, medium chain acyl-CoA dehydrogenases deficiency (MCADD) is the most common enzymatic defect.

The clinical manifestation depends on the underlying enzymatic defect and residual enzyme activity leading to a wide spectrum of symptoms. Although most FAOD are amenable to therapy, the clinical outcome depends on the severity of the specific defect. Cornerstones of treatment are the avoidance of fasting/catabolism and in long chain FAOD fat restriction accompanied by medium chain triglyceride supplementation [2, 3].

Since 2005, the FAO diseases MCAD-, LCHAD-/TFP-, VLCAD- and carnitine cycle defects are target diseases of the nationwide German newborn-screening program using the tandem mass spectrometry technique. This resulted in a considerable reduction of morbidity and mortality in patients suffering from FAOD. Newborn-screening also detects children with mild phenotypes or non-diseases. It remains unclear whether subjects with these mild (biochemical) phenotypes and/or novel genetic variants are at risk for the development of a clinically relevant phenotype.

With informative newborn-screening results, further confirmation is needed to specify defects of mitochondrial FAO. Specific enzyme assays are available for most fatty acid oxidation defects in order to determine residual enzyme activities [4]. Nevertheless, most of these assays are rather time consuming and technically ambitious with a risk of bacterial or fungal contamination [5]. The assays involve harvesting fibroblasts by skin biopsy. The culturing of fibroblasts under sterile conditions is laborious and time-consuming. The use of radioactive substances makes the investigation expensive and can only be carried out in specialized laboratories approved for handling radioactive materials.

Based on the work of Dessein et al. [6], the aim of our study was to optimize an ex vivo ´selective screening-assay` via tandem mass spectrometry for rapid and non-invasive confirmation of FAOD which allows discrimination of short chain acyl-CoA dehydrogenase- (SCAD-), medium chain acyl-CoA dehydrogenase- (MCAD-), very long chain acyl-CoA dehydrogenase-(VLCAD-), long chain hydroxyl acyl-CoA dehydrogenase- (LCHAD-) as well as carnitine transporter- (CTD-) defects.

An assay using end labeled deuterated acylcarnitine profiling has been developed to confirm the enzymatic defect by incubating human whole blood cells with stable end labeled palmitate.

Regarding potential metabolic risk stratification and the underlying genetic background, concentrations of specific acylcarnitines were compared between particular associated variants of these FAOD.

## Methods

Twenty-six healthy children and adolescents between 0 and 17 years of age (22 males, 4 females) undergoing elective surgical interventions served as control group. At the time of sample collection they had no intercurrent disease nor any identified metabolic defect.

The group of FAOD patients comprised 47 children (28 male, 19 female) with positive newborn screening results and confirmed diagnosis (SCAD-(*n* = 3), MCAD-(*n* = 30), VLCAD- (*n* = 6) and LCHAD- (n = 6) deficiency and primary carnitine transporter defect (*n* = 2). Usually, confirmation of the defect after informative newborn-screening was done by diagnostic DNA sequencing and/or enzymatic analysis.

This study was approved by the Ethical Review Board of Hannover Medical School (date: 21^st^ November 2008 Nr. 5176), informed consent was obtained from parents of controls, material from FAOD patients were investigated as routine work-up.

The method used here is based on the work by Dessein et al. [6], with minor modifications. All usual chemicals and liquids were purchased from Sigma Aldrich with the highest analytical quality (Deisenhofen, Germany). The solvents for mass-spectrometry were purchased from Biosolve BV (Valkenswaard, The Netherlands). The stable isotopes [16-2H3]-pamitoylcarnitine, [8-2H3]-octanoylcarnitine and [4-2H3]-butyrylcarnitine which were used as internal standards were obtained from Mr. H. ten Brink, VU Medical Center (Amsterdam, The Netherlands) distributed by EQ Laboratories GmbH (Augsburg, Germany). The substrate [16-2H3, 15-2H2]-palmitate was purchased from Labor Ehrensberger (Augsburg, Germany).

All control and patient samples were collected in EDTA-tubes (1–3 mL), stored at 4 °C and further processed within 24 h. Leukocytes were quantified automatically using a cell counter (Sysmex, Germany).

Sample preparation: Carnitine and [16-2H3, 15-2H2]-palmitate were dissolved in ethanol at a final concentration of 20 mM each. 10 μL of the stock solutions were pipetted into Eppendorf tubes, evaporated at 37 °C under nitrogen to dryness and kept at −20 °C before use. Deuterated acylcarnitines were dissolved in ethanol as internal calibrator solution (ISTD) containing calibrators d3-palmitoylcarnitine: 0.039 nmol, d3-octanoylcarnitine: 0.087 nmol and d3-butyrylcarnitine: 0.099 nmol.

Each whole blood sample was mixed carefully before use. 100 μL of whole blood were transferred into Eppendorf tubes containing carnitine and [16-2H3, 15-2H2]-palmitate, incubated in a shaker (Eppendorf, Hamburg, Germany) at 550 rpm and 37 °C for about 6 h. Samples were quenched by rapid freezing at −20 °C and stored. The samples were thawed at room temperature prior to the measurement. 1 mL of the internal standard solution (ISTD) was added to each tube and sonicated for 30 min. The samples were centrifuged (Eppendorf) for 10 min with 14.000 g. The clear supernatants were transferred into new Eppendorf-tubes and kept evaporated at 37 °C under nitrogen to dryness. 200 μL butanolic HCl was added to each tube, sealed and incubated at 65 °C for 15 min. Samples were dried at 65 °C under nitrogen and redissolved in 150 μL elution solvent (methanol/water 80:20, *v*/v).

For MS/MS analysis, a system consisting of a MICRO tandem mass spectrometer (Waters, Eschborn, Germany) equipped with a PAL autosampler and a 1525 μ gradient pump (Waters) was used. All measurements were done in ESI positive mode with a 3.3 kV capillary cone voltage and 30 V cone. The source temperature was 120 °C with a desolvation temperature of 350 °C. For analyzing acylcarnitines a precursor ion scan of m/z 85 (range m/z 200–550) was performed. 20 μL per sample was injected with a constant isocratic flow rate of 35 μL/min (solvent: acetonitrile/water 80:20 *v*/v). For the detection and quantification the MassLynx software 4.1 (Waters) was used. Run time per sample was two minutes at room temperature.

Each sample was incubated in independent double assay duplicates and measured twice. From all 4 measurements averages were calculated.

The statistical analysis was performed with Sigmaplot 12.5 (Systat software Inc., San Jose, California, USA) and Medcalc 17.0 (MedCalc Software bvba, Mariakerke, Belgium). To compare specific end-labeled acylcarnitine concentrations between controls and particular disease groups, the Mann-Whitney Rank Sum test was used. The comparison of MCADD variants was carried out using ANOVA. At a level of *p* < 0.05, a significant difference between groups was assumed.

## Results

For demographic data, genetic background and clinical phenotypes of our patient group see Table 1.

**Table 1:** Demographic data, genetic or biochemical background and clinical phenotypes [23]

Patient Group	Age [years] range	Biochemical findings or variants found	Number of Patients	Clinical course
*ACADM* (NM_000016.5): MCADD (19 boys, 11 girls)	0–17	c.985[A > G];[A > G] (Class 5; Class 5) c.985A > G(;)c.199 T > C (Class 5; Class 3) c.985A > G(;)c.362C > T (Class 5; Class 5) c.985A > G(;)c.717C > G (Class 5; Class 5) c.985A > G(;)c.476G > A (Class 5, Class 3) mutation diagnosis was not performed, diagnosis based on metabolites	19 3 1 1 1 5	All were detected by selective newborn screening, no metabolic decompensation or clinical abnormalities based on the MCADD defect occurred
*ACADVL* (NM_000018.3):VLCADD (2 boys, 4 girls)	0–4	c.848[T > C];[T > C] (Class 4; Class 4)	2	no metabolic decompensation or clinical abnormalities based on the VLCADD defect occurred
		c.848 T > C(;)c.1357C > T (Class 4; Class 5)	1	no metabolic decompensation or clinical abnormalities based on the VLCADD defect occurred
		c.538G > A(;)c.1367G > A (Class 4; Class 4)	1	infection associated CK elevation occurred
		c.779C > T(;)c.1700G > A (Class 4; Class 4)	1	infection associated CK elevation occurred
		mutation diagnosis was not performed, diagnosis based on enzymatic analysis and metabolites	1	cardiomyopathy and recurrent infection associated rhabdomyolysis occurred in this patient
*HADHA* (NM_000182.4): LCHADD (5 boys, 1 girl)	1–6	c.180 + 3A > G(;)c.1528G > C (Class 4; Class 5)	1	no metabolic decompensation or clinical abnormalities based on the LCHADD defect occurred
		c.1528[G > C];[G > C] (Class 5, Class 5)	1	recurrent infection associated CK elevation occurred
		c.914 T > A(;)c. 1528G > C (Class 3; Class 5)	3	One boy had no metabolic decompensation or clinical abnormalities based on the LCHADD defect, the younger brother had elevated CK levels and suffers from psychomotoric retardation. Another boy had no metabolic decompensation or clinical abnormalities based on the LCHAD defect
		mutation diagnosis was not performed, diagnosis based on enzymatic analysis and metabolites	1	diagnosis due to severe metabolic decompensation accompanied by organ failure at the age of 8 months while suffering from gastrointestinal infection
CTD (1 boy, 1 girl)	3–5	mutation diagnosis was not performed, diagnosis based on enzymatic analysis and metabolites	2	no metabolic decompensation or clinical abnormalities based on the CT defect
ACADS (NM_ NM_000017.3): SCADD (1 boy, 2 girls)	10–13	c.625[G > A];[G > A] (Class 1; Class 1)	2	one patient was clinically unremarkable except for obesity, one suffered from psychiatric problems
		mutation diagnosis was not performed, diagnosis based on metabolites	1	clinically unremarkable

### SCADD

**Fig. 1:**
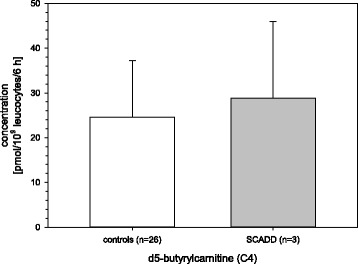
Butyrylcarnitine of SCADD patients versus controls

Butyrylcarnitine (C4) concentrations of SCADD patients were higher compared with controls (Fig. 1). However, the difference was not significant (*p* = 0.642). A significant increase of butyrylcarnitine was evident when compared with the other FAO diseases (*p* < 0.05) analyzed in this study.

### MCADD

**Fig. 2:**
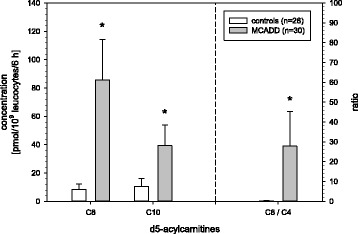
Medium chain acylcarnitines and the C4/C8 ratio of MCADD patients versus controls; * = significant difference *p* < 0.05

In samples of MCADD patients concentrations of octanoylcarnitine (C8), decanoylcarnitine (C10) as well as the ratio octanoylcarnitine (C8)/butyrylcarnitine (C4) were higher compared with the control population (Fig. 2). Acylcarnitines C8 and C10 were significantly (*p* < 0.05) increased in relation to the relevant acylcarnitines of controls. Ratios C8/C4 and C8/C10 were significantly increased in MCADD samples as well.

**Fig. 3:**
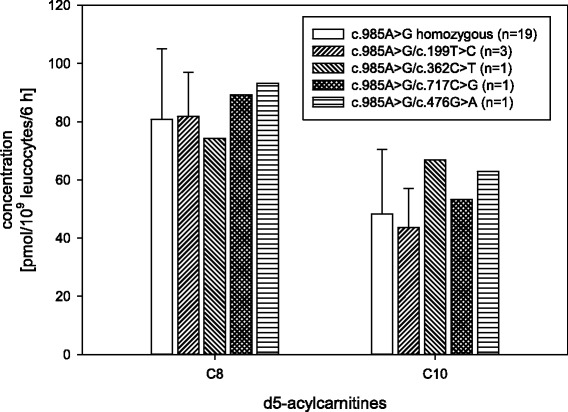
Medium chain acylcarnitines C8 and C10 in samples with various underlying MCADD variants

The comparison of ‘common’ homozygous to compound heterozygous variants of MCADD showed minor differences of C8 and C10 acylcarnitine concentrations (*P* = 0.542) (Fig. 3).

### VLCADD

**Fig. 4:**
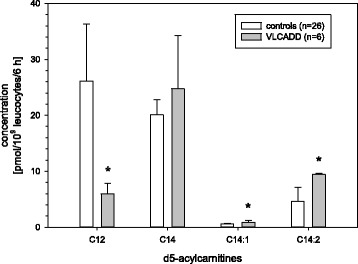
Long chain acylcarnitines in samples of VLCADD patients versus controls; * = significant difference *p* < 0.05

In samples of VLCADD patients dodecanoyl- (C12), tetradecenoyl- (C14:1) and tetradecadienoyl- (C14:2) carnitines differed significantly compared with controls (Fig. 4) and between FAOD groups. C12 was markedly reduced in VLCADD samples (*p* < 0.001), while C14:1 and C14:2 as well as ratios of C14:1/C12 and C14:2/C12 were significantly higher (*p* < 0.001). Control samples showed lower tetradecanoyl- (C14) concentrations, but the difference is not significant (*p* = 0.545). C14 was only discriminating between VLCAD patients and the FAO disease groups (*p* < 0.001).

**Fig. 5:**
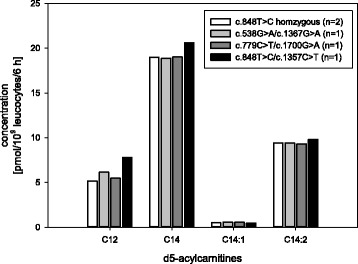
Long chain acylcarnitines in samples with various underlying VLCADD variants

Homozygous VLCADD variants showed slightly non-significant differences of relevant acylcarnitines in comparison to compound heterozygous variants (Fig. 5, due to small sample size no statistical analysis was performed).

### LCHADD

**Fig. 6:**
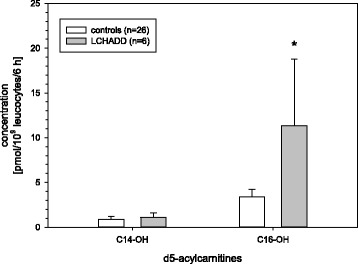
Hydroxylated long chain acylcarnitines in samples of LCHADD patients versus controls; * = significant difference *p* < 0.05

**Fig. 7:**
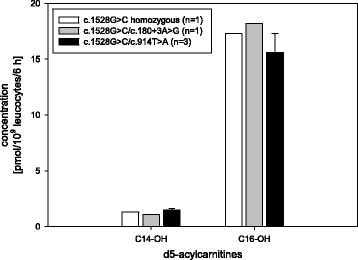
Hydroxylated long chain acylcarnitines in samples with various underlying LCHADD variants

The Hydroxylated acylcarnitines 3-hydroxy tetradecanoylcarnitine (C14-OH) and 3-hydroxy hexadecanoylcarnitine (C16-OH) were elevated in samples of LCHADD patients (Fig. 6) compared to controls. C16-OH levels were significantly higher (*p* < 0.05), while differences in C14-OH levels were not significant (*p* = 0.057). A significant increase of C14-OH levels was evident when compared with other FAO diseases of this study (*p* < 0.05). The differences of the hydroxylated acylcarnitines between the three different LCHADD variants were not significant (Fig. 7, due to small sample size no statistical analysis was performed).

### CTD

**Fig. 8:**
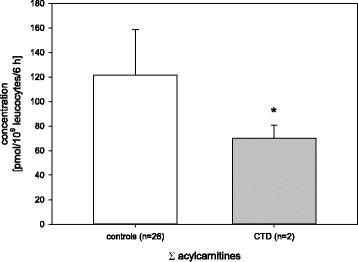
Sum of all determined acylcarnitines of CTD patients versus controls; * = significant difference *p* < 0.05

The sum of all acylcarnitines measured in control samples was significantly higher compared to samples of patients with CTD (p < 0.05) (Fig. 8). There was no significant difference compared to other FAO diseases tested (*p* = 0.226).

## Discussion

In contrast to the study by Dessein et al. [6] we focused on samples from children and adolescents. Additionally we included samples from patients suffering from LCHAD and carnitine transporter deficiency. The assay can reliably discriminate between healthy controls and different FAO patient groups (MCAD-, VLCAD-, LCHAD- and carnitine transporter deficiency) with disease-specific end-labeled acylcarnitine patterns. In 3 SCADD patients C4 carnitine levels were elevated in comparison to controls; probably due to small patient numbers no significance could be found.

This assay determines acylcarnitine patterns in vitro without using particular enzyme specific substrates; therefore residual specific enzyme activities cannot be directly estimated.

One advantage of this non-invasive assay is the use of an end-labeled non-radiochemical substrate. Thus, disease specific accumulating acylcarnitine patterns (non-metabolized substrate) can be detected. In many radiochemical flux assays, only metabolic end products are measured and further exploration is necessary to detect specific FAOD. The blood cells were loaded and thus stressed with palmitic acid, therefore, results should be more pronounced compared to acylcarnitine profiles derived from anabolic non-stressed patients.

Further advantages are easy handling, storage and transport of samples.

We compared end-labeled acylcarnitines in specific enzyme defects of FAO with different underlying disease causing variants.

Comparing the three MCADD missense variants (each compound heterozygous for c.985A > G, see Table 1) with the common homozygous c.985A > G variant, no significant difference in specific acylcarnitines and ratios could be found in vitro (see Fig. 3). This is in line with studies from two German groups [7, 8], who found no significant differences of in vivo acylcarnitine markers between patients homozygous for the c.985A > G variant and compound heterozygous patients for the c.985A > G variant in combination with variants other than c.199 T > C. This constellation probably has an intermediate effect on enzyme activity while other variants will probably impair MCAD activity comparable to the c.985A > G variant. With regard to residual enzyme activity, other groups found higher MCAD activities in vitro in most non-common variants potentially leading to a milder clinical course [9–11]. Of the four MCAD missense variants of our patients, the milder c.199 T > C variant displays residual octanoyl-CoA oxidation activities in the range of 22% to 47% in vitro [9]. The c.362C > T variant results in a considerably impaired MCAD enzyme activity in vitro [12], the c.476G > A and c.717C > G variants are of unknown clinical significance, to our knowledge no further data exist in the literature. Both variants could probably be classified as pathogenic as they affect both highly conserved areas of the *MCAD* gene, see Table 1. Finally, the assay described by us is not able to discriminate between different genetic variants in MCADD.

All MCADD patients were screened and diagnosed through newborn mass screening; the treatment was based on general recommendations to avoid prolonged fasting and carnitine supplementation only as required. Under this regimen, none of these patients suffered clinical symptoms or metabolic decompensations, see Table 1.

In VLCADD and LCHADD patients, no obvious difference between acylcarnitines in vitro and genotypes could be found as well. Due to low patient numbers, no statistical analysis was performed (see Figs. 5 and 7).

Two VLCADD patients were homozygous for the c.848 T > C missense variant whereas the c.848 T > C is known to be likely pathogenic resulting in a clinically milder disease with reduced penetrance due to residual enzymatic activity [13, 14]. One patient was compound heterozygous for this and the variant c.1357C > T, described as nonsense variant, to our knowledge no further data exists concerning clinical significance of this variant. One patient was compound heterozygous for the c.538G > A [14] and c.1367G > A [15] variants resulting in a VLCAD residual enzyme activity <10% in this case and the other one was compound heterozygous for the c.779C > T and c.1700G > A variants, both known as disease causing variants [15, 16]. As shown in Table 1, clinical courses of our VLCADD patients are not reflected in the in vitro acylcarnitine profiles or mutation analysis.

Concerning the LCHADD patients, one was homozygous for the common c.1528G > C variant, 4 patients were compound heterozygous for the common and other variants, see Table 1 [17–19]. As in other FAO diseases, in our LCHADD patients the clinical presentation could not be predicted from metabolite profiles in vitro or molecular genetic results. This fact is even more obvious in one family of our cohort with two affected boys carrying the same genotype presenting with different clinical courses, see Table 1. This seems to indicate that further epigenetic factors (variants of other genes, epigenetic factors, and others) could have an influence.

Treatment of all VLCADD and LCHADD patients was based on the consensus papers published in 2009 [2, 3].

Since no correlation between genotype and biochemical/clinical phenotype could be shown, especially in FAOD [7, 20–22], other biomarkers like organic acids in urine, acylcarnitines, free carnitine concentrations as well as clinical symptoms are used to predict the clinical course of FAOD patients. Other environmental and genetic factors may affect individual phenotypes and outcome. Clinical symptoms may differ remarkably within one family as shown in 2 LCHADD cases of our cohort (see Table 1).

We are aware of the limited patient numbers of our study especially in long chain FAOD. To corroborate our data, further patients should be investigated biochemically as well as genetically to correlate these and clinical data.

## Conclusion

The functional assay described here is less time consuming and relatively simple in comparison to other published methods and can be used to confirm patients suspected to suffer from MCADD-, VLCADD-, LCHAD- and carnitine transporter- deficiency. Different genotypes cannot be distinguished by acylcarnitine profiling in vitro.

## Abbreviations

CK: Creatine kinase
CTD: Carnitine transporter defect
FAO: Fatty acid oxidation
FAOD: Fatty acid oxidation disorder
LCHADD: Long chain hydroxy acyl-CoA dehydrogenase deficiency
MCADD: Medium chain acyl-CoA dehydrogenase deficiency
SCADD: Short chain acyl-CoA dehydrogenase deficiency
VLCADD: Very long chain acyl-CoA dehydrogenase deficiency
